# Mapping the Brain’s Glymphatic System

**DOI:** 10.3390/biomedicines14020409

**Published:** 2026-02-11

**Authors:** Konstantinos Voumvourakis, Nikolaos S. Thomaidis, Sotirios Tsiodras

**Affiliations:** 1 Attikon University Hospital, National and Kapodistrian University of Athens, Rimini 1, 12462 Athens, Greece; sotirios.tsiodras@gmail.com; 2 Metropolitan Hospital, 9 Ethn. Makariou & 1 El. Venizelou Str., 18547 Piraeus, Greece; 3 Laboratory of Analytical Chemistry, Department of Chemistry, National and Kapodistrian University of Athens, Panepistimiopolis Zographou, 15772 Athens, Greece; ntho@chem.uoa.gr

**Keywords:** glymphatic system, cerebrospinal fluid, interstitial fluid, aquaporin-4, perivascular spaces, blood–brain barrier, meningeal lymphatics, intramural periarterial drainage, amyloid-β, sleep

## Abstract

The glymphatic system is a fluid-transport framework in which cerebrospinal fluid (CSF) enters the brain along perivascular routes, exchanges with interstitial fluid (ISF), and exits toward venous, perineural, and meningeal lymphatic pathways enabling waste clearance. Recent studies have clarified the anatomical components that regulate solute movement. The perivascular astrocyte endfeet, which are enriched in polarized aquaporin-4 (AQP4) expression, create a high-permeability water interface that facilitates CSF–ISF exchange. Multiscale physical drivers such as cardiac pulsation, arteriolar vasomotion, and brain-state changes during sleep regulate the timing and efficiency of the glymphatic transport. A broad spectrum of solutes is transported through this pathway, from small metabolites to extracellular proteins including amyloid-β and tau, as well as exogenous tracers and some lipid-associated species. Glymphatic redistribution may interface with other clearance systems, including the brain-to-blood efflux via blood–brain barrier (BBB) transport, intramural periarterial drainage (IPAD) that clears along vascular basement membranes and the meningeal lymphatic pathways that drain macromolecules to deep cervical lymph nodes. These different routes may be interconnected and may represent a waste clearance network with complementary roles assigned to different mechanisms. Moreover, state dependence (notably sleep) and vascular health modulate glymphatic flux, offering plausible links between glymphatic system dysfunction, aging and neurodegeneration. Methodological advances—from intrathecal contrast magnetic resonance imaging (MRI) to *in vivo* two-photon imaging and tracer-kinetic modeling—have provided new insights into the anatomical scaffold and kinetics of the glymphatic system. Advances in glymphatic anatomy, together with growing evidence implicating glymphatic dysfunction in neurodegeneration, point towards a unifying framework that is urgently needed. Our synthesis spans glymphatic structure, fluid routing, and the repertoire of transported solutes and links to complementary clearance routes, supporting a unified model in which glymphatic clearance represents an important contributor of cerebral homeostasis. Understanding glymphatic dysfunction may guide the establishment of diagnostic imaging biomarkers that have the potential to assist in therapeutic modulation of neurodegenerative diseases.

## 1. Introduction

The central nervous system (CNS) is among the most metabolically active organs, consuming nearly 20% of total oxygen at rest and generating large quantities of metabolic byproducts, including lactate, reactive oxygen species, and aggregation-prone proteins such as amyloid-β (Aβ) and tau [1,2]. Efficient clearance of these metabolites and solutes from the interstitial extracellular space is essential to prevent toxicity and maintain brain homeostasis [3]. Moreover, clearance mechanisms sustain ionic balance and neurotransmission, both fundamental for neural activity and overall CNS function [4,5]. Additionally, impairment of waste clearance is increasingly recognized as a central element in the pathogenesis of several neurological disorders [3].

In peripheral tissues, waste clearance is efficiently carried out predominantly by the lymphatic system, which drains interstitial fluid (ISF) and solutes toward the lymph nodes. However, the brain poses a paradox; the parenchyma lacks conventional lymphatic vessels, apart from the recently characterized meningeal lymphatics that reside in the dura matter [6,7]. For decades, the absence of a classical lymphatic drainage system prompted investigations into alternative pathways capable of clearing waste from the brain.

Early observations established the dynamic circulation of cerebrospinal fluid (CSF) through the ventricles and the subarachnoid spaces, as well as an exchange of solutes between the CSF and the cerebral extracellular space [8]. These studies suggested a role for CSF in the waste clearance process via perivascular routes, yet the precise anatomical pathways mediating CSF–ISF exchange remained elusive. Only in the past decade have advances in *in vivo* imaging and tracer kinetics clarified the pivotal role of CSF in perivascular clearance processes [3].

The present review synthesizes current knowledge on the anatomy and physiology of the glymphatic system, a glia-dependent perivascular network facilitating CSF-ISF exchange and interstitial solute clearance. The following sections delineate the structural components that regulate glymphatic transport, including perivascular spaces (PVS) and aquaporin-4 (AQP4) water channels, describe the variety of solutes conveyed through these routes and discuss the interface between glymphatic flow and complementary clearance mechanisms. Finally, we propose an integrated view of brain waste removal, emphasizing the interdependence of perivascular, lymphatic and barrier-mediated pathways in maintaining cerebral homeostasis.

## 2. The Glymphatic System

Assessment of glymphatic function has been facilitated through the development of a variety of techniques that employ imaging and tracer kinetics. In experimental rodent models, these involve intracisternal or intrathecal tracer infusion of fluorescent or magnetic resonance imaging (MRI) contrast agents, followed by time-lapse or two-photon microscopy to detect tracer influx and efflux *in vivo* and *ex vivo* [9].

Iliff et al. (2012) first demonstrated that fluorescent tracers infused into the cisterna magna enter the brain along periarterial spaces and exit along perivenous routes [10]. These experiments revealed a directed convective movement of CSF through the parenchyma, suggesting the presence of a glial-dependent transport mechanism coupling CSF flow to interstitial solute clearance. The dependence on a glial cell type and the functional similarity to the lymphatic transport led tο the designation of this system as the “glymphatic system”.

Using fluorescent tracers and *in vivo* imaging, Xie et al. (2013) demonstrated that the glymphatic activity is state-dependent, with the interstitial space volume expanding by ~60% during natural sleep or anesthesia [11]. The CSF–ISF exchange was enhanced, and Aβ clearance was accelerated during sleep, while in the awake state, elevated noradrenergic tone suppressed glymphatic transport. In contrast to passive diffusion, the glymphatic system operates mainly through convective fluxes, driven by multiple physiological forces including arterial pulsatility, slow vasomotion and respiratory cycles [12,13]. These dynamic factors generate oscillating perivascular pressure gradients that propel CSF into the brain parenchyma and facilitate ISF efflux toward perivenous spaces [7].

Whole-brain imaging in rodents using dynamic contrast-enhanced MRI provided evidence for a brain-wide glymphatic pathway. Two studies from the same research group demonstrated the periarterial CSF influx of intrathecally injected contrast agents and molecular size-dependent CSF-ISF exchange, with subsequent efflux along perivenous spaces [14,15]. Supported by complementary optical microscopy data, these imaging studies provided the first evidence for a coordinated, global anatomic framework of the glymphatic system.

Intrathecal administration of gadolinium-based contrast agents and T1-weighted imaging was first employed to detect CSF flow in humans to directly assess glymphatic function. However, this method is considered invasive and presents some limitations for clinical routine use [16]. Development of non-invasive methods based on diffusion-weighted MRI has therefore dominated studies that aimed to assess glymphatic function in humans. The most used method is diffusion tensor imaging along the perivascular space (DTI-ALPS) index, which quantifies directional water diffusivity along perivascular spaces (PVS) [17]. This outcome serves as a proxy for indirectly inferring glymphatic function. The findings from these studies have further provided supporting evidence for a functional glymphatic system in humans.

This conceptual breakthrough supported a glymphatic concept across species, indicating mechanistic and translational continuity from rodents to humans. The proposition of a dynamic, glia-dependent clearance network has redirected our experimental focus toward unraveling waste clearance networks that contribute to CNS homeostasis.

## 3. The Anatomy and Composition of the Glymphatic System

**Figure 1:**
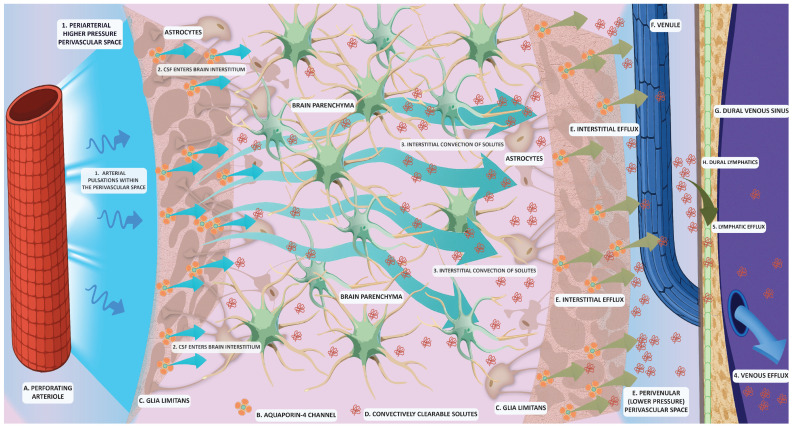
Principles of classical glymphatic physiology. (1) Perforating arteriole (A) pulsation provides the driving force for the arterial perivascular space cerebrospinal fluid (CSF) to enter (2) the parenchymal interstitium. Aquaporin 4 channels (B) of the *glia limitans* (C) enable CSF entry into the parenchymal interstitium and subsequent exchange with the interstitial fluid (ISF) by increasing the water permeability of perivascular astrocytic endfeet. Pressure gradients result in bulk flow convective currents of interstitial fluid (3) that enable the clearance of solutes (D) towards the lower-pressure perivascular space (E) of the venules (F). Solutes are ultimately cleared away through the venous (4) and lymphatic (5) efflux through the dural venous sinuses (G) and their attending dural lymphatic vessels (H).

According to the prevailing model of glymphatic anatomy, CSF enters the brain along the periarterial PVS. These periarterial compartments are ensheathed by astrocytic endfeet enriched with AQP-4 channels, which enable water flux across the astrocytic membrane and facilitate CSF-ISF exchange. The resulting CSF–ISF admixture is then directed toward perivenous spaces, ultimately draining into meningeal and cervical lymphatic structures (Figure 1) [18,19]. Additionally, recently identified lymphatic vessels in the dura mater provide a structural bridge between perivascular glymphatic clearance and extracranial lymphatic drainage [20]. Therefore, the glymphatic flow is structurally dependent on the volume of the PVS and the polarization of AQP4 channels, features that are further discussed in detail.

### 3.1. Perivascular Spaces (PVS)

Virchow–Robin spaces are pial-lined, ISF-filled compartments that line penetrating cerebral arterioles, capillaries and venules as those vessels traverse through the brain parenchyma [21]. PVS are located at predictable anatomical locations (e.g., the cortex and basal ganglia), and their dimensions vary with age, vascular risk factors, as well as disease state [22,23]. Each space comprises an endothelial vessel wall surrounded by a pial sheath and an outer boundary formed by the astrocytic endfeet densely populated with AQP4 channels underlain by the basal lamina of the *glia limitans* [24]. Perivascular basement membranes formed by endothelial and glial extracellular matrix components are key structural elements that delineate the trajectory of the glymphatic flow [25].

The role of PVS extends beyond facilitating convective fluid movement and waste clearance. Their topology and CSF-ISF flux patterns provide pathways for the distribution of nutrients, neuromodulators and growth factors across brain regions, thereby supporting metabolic and ionic homeostasis [26,27].

Immune cells that accumulate in PVS during neuroinflammatory states can exit the CNS along glymphatic flow toward the deep cervical lymph nodes, linking central immune surveillance to the meningeal lymphatic system [28]. During physiological conditions, this process promotes immune monitoring, while in pathological contexts, it facilitates the removal of immune cells from the inflamed parenchyma [29]. This notion is supported by findings showing the presence of immune cells and axonal antigens in the cervical lymph nodes in several CNS disorders [21]. Burden of enlarged PVS (ePVS) documented via MRI is also used as a surrogate marker of glymphatic dysfunction [30]. Of note, enlarged PVS have been reported in patients with Alzheimer’s disease (AD) [31], dementia [32] and multiple sclerosis [22], among other conditions. Dysregulation of the PVS-lymphatic system axis and increased immune trafficking to cervical lymph nodes may thus represent converging mechanisms in neurodegenerative pathophysiology.

The PVS compartment also accommodates vascular pulsations that drive glymphatic flow. Arterial pulsations within the central lumen generate perivascular fluid propulsion [33]. Alterations in vessel compliance, such as those induced by hypertension, impair perivascular pumping and can lead to stagnation of the glymphatic efflux through the PVS [12]. Such dysfunction ultimately compromises waste clearance and parenchymal homeostasis.

Overall, PVS constitute the fundamental anatomic substrate of the glymphatic system, coupling vascular dynamics to interstitial equilibrium. The interplay among mechanical drivers, astrocytic water channels and immune elements supports proteostasis and interstitial stability. Disruption of any of these components may compromise solute clearance, thus potentially leading to neurodegeneration [21,33].

### 3.2. Astrocytic Endfeet and AQP4 Water Channels

Astrocytic endfeet refer to the expanded terminal processes of astrocytes that form a continuous sheath around cerebral blood vessels in the brain, positioning these glial cells at the interface between the vascular compartment and the brain parenchyma [7]. Through this close association, the astrocytic endfeet regulate capillary permeability, contributing to blood–brain barrier (BBB) integrity while also surrounding PVS and modulating CSF-ISF exchange [34]. This dual positioning renders astrocytes as key regulators of glymphatic transport and, subsequently, cerebral homeostasis [7].

The endfeet facing the PVS are densely enriched in AQP4, the predominant astrocytic water channel in the CNS. Polarized localization of AQP4 at the vascular endfeet membrane leads to the formation of dense orthogonal arrays that enable bidirectional water flux [25,35]. Proper anchoring of AQP4 depends on the interactions between adaptor proteins, the astrocytic cytoskeleton and the extracellular matrix. Disruption of these interactions results in AQP4 mislocalization or depolarization [25,36].

The subcellular distribution of AQP4 is dynamically regulated. Phosphorylation events and cytoskeletal remodeling can shift its polarization under physiological or stress conditions [37], allowing adaptive modulation of water transport and coupling between perivascular CSF flux and parenchymal ISF flow [35,36]. Experimental studies in rodents have demonstrated that genetic deletion of AQP4 leads to an approximately 70% reduction in tracer influx from the CSF into the brain parenchyma and severely impairs solute clearance [10,36]. Similarly, pharmacological inhibition of AQP4—for example, with the use of the small molecular inhibitor TGN-020—diminishes glymphatic flow and promotes accumulation of neurotoxic proteins in the parenchyma [38].

Disruption of AQP4 anchoring to the dystrophin-associated complex or to basal lamina proteins such as agrin, through deletion of *Dmd*, *Snta1* or related genes, results in AQP4 mislocalization [35]. Notably, *Snta1* deletion increases Aβ deposition and impairs glymphatic clearance *in vivo*, suggesting a role of AQP4 polarization in AD [39]. Moreover, AQP4 depolarization induced by LRRK2-mediated phosphorylation reduces clearance of interferon—γ, implicating the glymphatic system in the neuroinflammatory burden of Parkinson’s disease (PD) [40]. Loss of AQP4 perivascular polarization has also been observed with aging [41] and across diverse neuroinflammatory and neurodegenerative disorders [35,42,43,44].

Collectively, the efficiency and polarization of AQP4 in the vascular–glial interface seem to be crucial for maintaining effective glymphatic transport. Loss or mislocalization of AQP4 reduces water permeability, thus disrupting pulsatility- and pressure-driven coupling between perivascular CSF flow and parenchymal ISF, thus impairing waste clearance. This inability could promote the accumulation of neurotoxic metabolites and disturb ionic and osmotic equilibrium.

## 4. Process of Glymphatic Flow

The principal source of CSF, in mammals, is considered to be the choroid plexus epithelium in a process involving water transportation into the ventricular system [45]. Unidirectional ion transport drives isotonic water secretion across the epithelium, generating the bulk CSF that fills the ventricular system and the subarachnoid space [46] with motile cilia on ependymal cells facilitating CSF movement through the ventricles [47]. However, findings from experiments neutralizing water channels responsible for water transport into the ventricular system have challenged this theory, suggesting that extrachoroidal sites account for most or all of the CSF production [48]. Regardless, it is generally believed that choroid plexi account for most of the CSF production while extrachoroidal sites contribute a smaller portion [48]. Additionally, CSF production is not stable and tends to peak during nighttime [49].

Live two-photon imaging with intracisternal fluorescent tracers has shown that subarachnoid CSF enters the brain parenchyma along periarterial routes [10]. After entering these pathways, CSF exchanges with ISF within the parenchyma. Brain ISF arises primarily at the microvascular interface through transendothelial water and solute exchange across the BBB, and the endothelial transport defines both its volume and composition [50]. Convective flow between CSF and ISF enables solute transport deep within the parenchyma [10]. This process is modulated by brain state; during natural sleep or specific anesthetic conditions, the interstitial volume expands by approximately 60%, enhancing CSF-ISF convective exchange and solute clearance [11]. Conversely, human studies confirm the suppression of glymphatic function following sleep deprivation [51].

The timing and efficiency of glymphatic flow reflect the interplay of multiple physiological drivers. Particle-tracking velocimetry in rodents demonstrated that the CSF movement within PVS is pulsatile and tightly linked to the cardiac cycle [12]. In humans, real-time MRI has revealed that CSF flow also correlates with respiration, highlighting a respiratory contribution to craniospinal CSF dynamics [52]. Two-photon microscopy in awake mice further showed that low-frequency vasomotion, governed by smooth muscle activity, drives perivascular clearance, whereas suppressed vasomotion stalls solute transport [53]. Sleep-related global oscillations have likewise been implicated in glymphatic facilitation [54], and recent work has identified norepinephrine-mediated slow vasomotion as a key mechanism underlying sleep-enhanced clearance [55].

The perivascular astrocytic endfeet are densely packed with orthogonal arrays of AQP4 water channels that form specialized, highly permeable membrane domains at the interface between CSF and the interstitial space, allowing rapid transmembrane water exchange along vessel walls [56]. Polarized localization of AQP directs CSF-ISF flow along vascular trajectories, effectively operating as a unidirectional valve that promotes efficient fluid movement [35]. Distinct AQP4 protein isoforms display selective permeability, suggesting that these channels not only regulate water flux but also influence the access of macromolecules to the parenchyma [57].

Following CSF-ISF exchange and solute loading, efflux proceeds toward dural meningeal lymphatic vessels, which absorb brain-derived macromolecules and drain them to deep cervical lymph nodes [58]. Non-invasive intrathecal MRI studies in humans corroborate this macro-scale drainage route via meningeal lymphatics, showing age-related aberrations [59,60] consistent with declining glymphatic function [61]. Lymphatic vessels in the basal dura and adjacent to the subarachnoid space have also been implicated in CSF macromolecule clearance [62]. Collectively, meningeal lymphatics act as the distal outlet for glymphatic efflux, channeling solutes toward cervical lymphatic basins.

## 5. Interaction of Waste Clearance Systems

Experimental evidence links the glymphatic system to other cerebral waste clearance mechanisms, suggesting an integrated, multilayered network. Although direct *in vivo* validation remains limited, emerging evidence suggests that the glymphatic pathway may be functionally linked to other clearance networks, including the BBB-mediated efflux pathway, the intramural periarterial drainage (IPAD) pathway, the arachnoid granulations and cellular degradation mechanisms.

The BBB forms a selective interface between circulation and brain parenchyma, maintaining ionic balance within the ISF and restricting the entry of circulating factors that may trigger inflammatory cascades [63]. The integrity of the BBB depends on coordinated signaling between endothelial cells and astrocytes; any disruption of this interaction can provoke neuroinflammatory and neurodegenerative pathology [64]. Efflux transporters such as endothelial low-density lipoprotein receptor–related protein-1 (LRP1) and P-glycoprotein serve as principal routes for Aβ clearance across the BBB [65,66]. The glymphatic flow, by facilitating bulk ISF movement, redistributes solutes toward vascular surfaces, enabling BBB-mediated removal [61]. Enhanced convective flow and increased interstitial volume fraction during sleep [67] may therefore augment Aβ delivery to endothelial transporters, indirectly boosting BBB-dependent clearance [68]. Thus, BBB efflux and glymphatic redistribution may operate as complementary components of a coordinated waste-removal system.

The IPAD pathway relies on the vasomotor activity of smooth muscle cells to propel solutes along the basement membranes of cerebral capillaries and arteries [69]. In cerebral amyloid angiopathy, fluorescent dextrans have been shown to be cleared preferentially along these intramural routes, rather than via perivenous spaces [70]. Electron microscopy indicates that glymphatic periarterial influx and the IPAD efflux form contiguous, layered channels around the same vessels [71]: the glymphatic component delivers CSF into the parenchyma, whereas the IPAD pathway directs solute drainage along basement membranes within the tunica media. Experimental blockage of IPAD, such as after subarachnoid hemorrhage, impairs solute clearance without affecting glymphatic influx [72], reinforcing the notion that these two systems could share anatomical features yet are functionally distinct.

Arachnoid granulations have traditionally been viewed as CSF drainage portals [73]. Modern microscopy suggests they function as porous, filter-like structures at the interface between CSF and venous circulation. Their location adjacent to meningeal lymphatic channels implies potential co-functionality [74]. Together, arachnoid granulations and lymphatic conduits may form a dual outflow system handling both fluid filtration and solute transport [75].

Cellular degradation pathways provide an additional layer of clearance. Enzymatic mechanisms, such as neprilysin-mediated proteolysis, reduce extracellular Aβ burden; down-regulation of neprilysin elevates Aβ levels *in vivo* [76], whereas neprilysin gene transfer diminishes plaque load in experimental AD models [77]. Similar results have been reported following insulin-degrading enzyme deficiency [78]. Microglia employ digestive exophagy through lysosomal synapses to degrade Aβ depositions [79], while autophagy in pericytes mitigates α-synuclein accumulation [80]. These processes collectively lower the interstitial load of neurotoxic proteins, thus potentially indirectly complementing glymphatic, BBB, and IPAD-mediated clearance. In this hypothesis, they would form an interdependent defense network against proteostatic stress.

Overall, an interconnectedness between the glymphatic system and other clearance mechanisms may be speculated, with complementary roles assigned to different mechanisms. These possible interactions add complexity to the research efforts to clarify the waste clearance network of the brain; therefore, further research into this topic is warranted.

## 6. Spectrum of Transported Solutes

The glymphatic system mediates the movement and elimination of a wide range of solutes within the CNS. Solute clearance via the glymphatic pathway is proposed to occur through periarterial CSF-ISF exchange and preferential perivenous efflux, with solute transport mediated by a combination of diffusion, dispersion, and fluid-driven advection [5,7]. Enrichment of astrocytic perivascular endfeet with AQP4 leads to an increase in water permeability, thus facilitating CSF flow and subsequent exchange. A variety of solutes may be transported, including metabolic byproducts, neurotoxic proteins, physiological macromolecules, lipids, exogenous tracers and nucleic acid fragments. This diversity underscores its role in maintaining homeostasis and its potential contribution to neurodegenerative disease when impaired. Clearance efficiency varies by solute size and physicochemical properties, while other pathways, particularly BBB efflux, act synergistically [68].

Metabolic byproducts such as lactate and glucose derivatives are efficiently transported via glymphatic flow [81,82]. In rodents, lactate clearance is enhanced during sleep and diminished during wakefulness. Highly diffusible metabolites generated as metabolic waste of the brain, such as urea, may also be transported through the glymphatic system, though passive diffusion across the BBB likely predominates for such small solutes [3].

Neurotoxic proteins, including Aβ and tau, are among the best-characterized glymphatic cargos. In pivotal experiments, fluorescently labeled Aβ peptides injected into the interstitium were cleared preferentially along perivascular routes [10]. Glymphatic dysfunction due to AQP4 depolarization significantly reduces Aβ elimination [39,83], while similar impairment promotes Tau accumulation, including pathogenic phosphorylated variants relevant to neurodegeneration [38,84,85]. Because the glymphatic efflux is solute size-dependent [86], smaller Aβ fragments (~4 kDa) are cleared more readily than larger species such as tau oligomers and fibrils [87]. Particularly for Aβ, LRP1-mediated BBB efflux may further enhance clearance through combined convective and transporter mechanisms [68].

Beyond pathological aggregates, glymphatic transport distributes physiological proteins including apolipoprotein E (ApoE) and α-synuclein. CSF-derived human ApoE exhibits isoform-specific parenchymal uptake (ApoE2 > ApoE3 > ApoE4) and rapid diffusion through interstitial pathways [57]. In experimental PD models, the genetic deletion or pharmacologic inhibition of AQP4 reduces α-synuclein clearance, confirming its dependence on glymphatic transport [88,89].

Glymphatic circulation also contributes to nutrient and signaling molecules delivery. Two-photon imaging confirms the perivascular movement of small lipophilic compounds [90], while AQP4 depolarization in PD-like models increases lipid retention, suggesting impaired clearance [91]. Further evidence supports glymphatic involvement in the transportation of neurotransmitters, amino acids [92], growth factors and other neuroactive substances [27]. Ion flux between neighboring neurons via volume transmission may likewise be modulated by glymphatic flow [93].

Although anatomically distinct, the meningeal lymphatic and glymphatic systems are functionally interconnected [94]. Their interaction is evident from the direct CSF-ISF intermix as well as from the immune cell signaling within the meninges during homeostasis [94]. Meningeal lymphatics actively participate in CSF drainage [28], implying that soluble antigens and macromolecules exchange between the parenchyma and peripheral immune circuits. This integration enables CNS immune surveillance by coupling antigen transport to lymphatic communication [95].

Intrathecal administration of exogenous tracers has been instrumental in characterizing glymphatic transport capacity. Small gadolinium-based agents move efficiently along glymphatic routes, visualizing CSF-ISF exchange *in vivo* [10,15]. Both hydrophobic and hydrophilic solutes can traverse perivascular channels [96], although the molecular size imposes constraints [86]: large molecules (~40 kDa) experience restricted movement due to narrow astrocytic junctions [97], limiting their interstitial diffusion and efflux. Consequently, therapeutic antibodies and other macromolecular drugs may show limited glymphatic permeability, while smaller solutes, such as growth factors, are rapidly transported. Furthermore, the molecular shape and the charge of the solute may influence penetration even more strongly than the size of the solute [96]. Thus, the glymphatic system may function as a size- and property-selective conduit that could inform strategies for intrathecal drug delivery [98].

## 7. Conclusions and Clinical Implications

Recent evidence has shifted the field from a purely anatomical description of PVS to a process-oriented understanding of fluid and solute dynamics within the brain. The CSF and ISF movement, their intraparenchymal exchange and eventually efflux are governed by multiple physiological parameters and are coordinated across several clearance systems. Within this integrated framework, the glymphatic pathway contributes both to waste elimination and to solute redistribution, functioning in concert with other mechanisms such as the BBB transport, the IPAD pathway and the meningeal lymphatic outflow. The growing recognition of glymphatic interactions with diverse CNS pathways provides a plausible explanation for the disproportionate parenchymal solute accumulation and neurotoxic burden seen following perturbations in any single component, such as reduced vasomotion with vascular aging [23], loss of AQP4 polarity, sleep disturbances or impaired BBB efflux.

Despite substantial progress in characterizing the anatomy and function of the glymphatic system, critical questions remain and raise significant debate. The central role of AQP4 in bulk flow convection is challenged by studies employing AQP4 knockout experimental models supporting a predominant parenchymal diffusion rather than an AQP4-mediated convective transport [99]. Moreover, theoretical models emphasize that the hydraulic resistance of brain tissue argues against a homogeneous, high-magnitude convective flow throughout the parenchyma [100]. Additionally, methodological aspects, such as the use of tracer under pressure or anesthesia in pre-clinical models, may transiently overestimate actual bulk flow [101], while tracer kinetics can be consistent with mixed mechanisms encompassing both diffusion and convective flow [102], pinpointing toward solute efflux via both bulk flow and diffusion driven by the solute size [103]. Therefore, the importance of the glymphatic system should be interpreted with caution, acknowledging ongoing controversies, while emerging evidence is anticipated in the future.

Even if glymphatic dysregulation is increasingly highlighted in pre-clinical models of neurodegenerative diseases, including AD [104], traumatic brain injury [84] and PD [89], another topic of controversy is the translatability between rodents and humans. Key structural and physiological differences across species do not allow for a complete linear translation between the rodent and human brain [105]. Rodents demonstrate higher AQP4 perivascular polarization compared to humans [106], while humans exhibit higher anatomical complexity of PVS, allowing for more tracer influx [107]. Additionally, differences in the size and molecular profile of astrocytes between humans and rodents [108], as well as differences in the function of lymphatic vessels between species [109] are among the variations that underscore the need to interpret the experimental findings from rodents with caution. Recent studies have assessed glymphatic function in patients with neurodegenerative diseases [110,111,112]. A lower DTI-ALPS index in patients compared to either age- and sex-matched individuals or to patients with prodromal neurodegenerative phenotypes has been shown collectively. These observations point toward reduced water diffusion along PVS in patients with neurodegenerative conditions, indirectly indicating glymphatic dysfunction. Regardless, the translation of findings from rodents cannot be completely established, since the interpretation of outcomes must consider the controversies raised by species differences. Therefore, extensive research is warranted to fully delineate the unique anatomical and structural features of the human brain that will allow us to assess glymphatic function in the context of neurodegeneration.

This review sought to highlight mechanisms and interactions that may guide future exploration in this direction. As research in this field continues to expand, glymphatic function assessment may become an important component of diagnostic and prognostic evaluation in disorders characterized by abnormal protein aggregation, such as AD and PD. Future experimental work could target the development of minimally invasive real-time trackers that will enable the rapid visualization of flow dynamics across the whole brain, while such trackers should be able to represent glymphatic flow under awake state as well [7,27]. Such findings could further provide robust evidence concerning the actual bulk flow versus the diffusion rate. Beyond its proposed role in metabolic waste clearance, future studies on the glymphatic system should clarify its potential for distributing electrolytes, macromolecules, and other compounds that may enter the brain preferentially through the blood-CSF barrier at the choroid plexus. Evaluating the trajectories of glymphatic flow could reveal possible interactions between waste clearance mechanisms within the brain parenchyma and delineate the draining patterns. Unraveling of glymphatic flow dynamics may open new perspectives in the delivery of therapeutics, including large agents and oncologic drugs, within the CNS [7,98].

Recently developed MRI-based techniques offer non-invasive tools for quantifying glymphatic transport and assessing clearance efficiency *in vivo* [16]. The development of improved, safe, reproducible and minimally invasive diagnostic tools would greatly assist in detecting individuals with an early or accelerated decline in glymphatic clearance. Therefore, these techniques hold promising potential for patient stratification, prognosis of impaired clearance, and therapeutic monitoring. In parallel, targeted interventions aimed at improving sleep quality and restoring vascular pulsatility or vasomotion may represent novel approaches to enhance glymphatic performance and mitigate neurodegenerative risk.

## Data Availability

No new data were created or analyzed in this study. Data sharing is not applicable to this article.

## Abbreviations

The following abbreviations are used in this manuscript:

Aβ	amyloid-β
AD	Alzheimer’s disease
ApoE	apolipoprotein E
AQP4	aquaporin-4
BBB	blood–brain barrier
CNS	central nervous system
CSF	cerebrospinal fluid
IPAD	intramural periarterial drainage
ISF	interstitial fluid
LRP1	lipoprotein receptor–related protein-1
MRI	magnetic resonance imaging
PD	Parkinson’s disease
PVS	perivascular spaces
